# Nanomedicine for the Treatment of Viral Diseases: Smaller Solution to Bigger Problems

**DOI:** 10.3390/pharmaceutics16030407

**Published:** 2024-03-16

**Authors:** Suvankar Ghorai, Harshita Shand, Soumendu Patra, Kingshuk Panda, Maria J. Santiago, Md. Sohanur Rahman, Srinivasan Chinnapaiyan, Hoshang J. Unwalla

**Affiliations:** 1 Department of Cellular and Molecular Medicine, Herbert Wertheim College of Medicine, Florida International University, 11200 SW 8th Street, Miami, FL 33199, USA; sghorai@fiu.edu (S.G.); kpand014@fiu.edu (K.P.); msant206@fiu.edu (M.J.S.); mdsrahma@fiu.edu (M.S.R.); schinnap@fiu.edu (S.C.); 2 Department of Microbiology, Raiganj University, Raiganj 733134, India; harshitashand16@gmail.com (H.S.); patrasoumendu1111@gmail.com (S.P.); 3 Department of Chemistry and Biochemistry, Florida International University, 11200 SW 8th Street, Miami, FL 33199, USA

**Keywords:** HIV/AIDS, nanomedicine, nano vaccine, RNA virus, nano-herbal

## Abstract

The continuous evolution of new viruses poses a danger to world health. Rampant outbreaks may advance to pandemic level, often straining financial and medical resources to breaking point. While vaccination remains the gold standard to prevent viral illnesses, these are mostly prophylactic and offer minimal assistance to those who have already developed viral illnesses. Moreover, the timeline to vaccine development and testing can be extensive, leading to a lapse in controlling the spread of viral infection during pandemics. Antiviral therapeutics can provide a temporary fix to tide over the time lag when vaccines are not available during the commencement of a disease outburst. At times, these medications can have negative side effects that outweigh the benefits, and they are not always effective against newly emerging virus strains. Several limitations with conventional antiviral therapies may be addressed by nanotechnology. By using nano delivery vehicles, for instance, the pharmacokinetic profile of antiviral medications can be significantly improved while decreasing systemic toxicity. The virucidal or virus-neutralizing qualities of other special nanomaterials can be exploited. This review focuses on the recent advancements in nanomedicine against RNA viruses, including nano-vaccines and nano-herbal therapeutics.

## 1. Introduction

A retrovirus is a kind of RNA virus that modifies a host cell’s genome by introducing a DNA copy of its RNA genome into the cell’s DNA. The gag, pol, and env segments of the single-stranded RNA genome, which encode the structural and functional protein of the virus, have been identified in two copies of the retrovirus encased by a glycoprotein envelope. The advanced therapeutic technology has successfully treated and eradicated potentially fatal viral illnesses like smallpox and poliomyelitis thanks to technological advancements and mass vaccination approaches. Nonetheless, viral infections persist in placing a heavy strain on the international health structure in the present day [1]. There is an ongoing battle where scientists and healthcare providers frequently must compete to develop effective management plans for recently altered or emerging virus strains [2]. Vaccination is the most commonly utilized clinical technique for eliminating viruses, whereby viral antigens are administered to help bestow immunity. Although vaccinations against diseases like measles and hepatitis have been shown to be effective, numerous obstacles stand in the way of the general use of this approach against emerging viral kinds [3]. For instance, during each round of viral replication, the human immunodeficiency virus (HIV) exhibits a high rate of mutation as well as recombination [4]. Nine kilobases of the HIV genome encode for sixteen different proteins, including regulatory (Tat and Rev), auxiliary (Nef, Vif, Vpu, and Vpr), and important structural (Gag, Pol, and Env) proteins. HIV variations often predict how quickly the disease progresses and how the body reacts to antiretroviral therapy (ART). The high rate of mutation is due to poor proof-reading potentiality and error-prone HIV reverse transcriptase (RT) [5]. The principal reason for HIV’s enormous genetic diversity is the viral envelope (Env) glycoprotein’s extraordinary rate of variability, which ironically makes it the prime target of neutralizing antibodies [6]. It can elude the effects of neutralizing antibodies as well as supplemental immune responses because of the rapid rate of mutation, which ranges from 1 to 10 mutations per genome every cycle of replication, broad plasticity in conformation, and considerable shielding of the glycan in surrounding tissue [7]. Another drawback in vaccine advancement is the vast gene variety in viruses. For example, HIV has four groups: HIV: P (pending), N (non-M/non-O), O (outlier), along with M (major). Group M is composed of nine subtypes or clades denoted by the letters A to K. Research has demonstrated that amino acid differences can range from 30% within subtypes to 42% between subtypes. These changes in amino acids are due to changes in genome sequence varying among the subtypes [8]. The difficulty of developing a universal vaccine is increased by the actuality that 10–20% of HIV-infected people in certain African nations have two or more viral variants (subtypes and recombinant forms) that are common in these places [9]. The absence of suitable animal models, inadequate knowledge of the correlates of immune protection, and the pharmaceutical industry’s meagre funding are additional obstacles to HIV vaccine development [10]. The public has been greatly alarmed by recent viral outbreaks in the past few decades, which have been brought on by the coronaviruses, Ebola, Zika, and influenza. These outbreaks have also brought attention to the lack of systems and tactics that can be used for quickly countering new pandemics. It is crucial to create widely pertinent therapeutic approaches which can be quickly implemented in countering infections caused by viruses [11]. Medications which obstruct several phases in the life cycle of a virus are used in common therapeutic techniques against viruses [12]. While antiretrovirals might be fairly successful, they need strict patient biddability and may result in negative consequences and side effects like nausea, diarrhea, lipodystrophy, and skin problems [13]. Developments in nanotechnology can potentially aid in overcoming these challenges and present fascinating chances for growth by providing innovative, all-around nanotherapeutic platforms to treat viral infections. Drug-loaded nanoparticles (NPs) can reduce systemic toxicity and improve pharmacokinetics and pharmacodynamics [14]. Certain nanomaterials can also kill viruses directly because of their inherent antiviral properties [15]. In the recent past, infections have been selectively bound to and neutralized by NPs that have undergone functionalization and manipulation.

## 2. Retroviruses: The Overview

The *Retroviridae* family of retroviruses only infects vertebrates and is responsible for several illnesses, such as cancer and AIDS [16]. Retroviruses replicate only by a specific enzyme called reverse transcriptase (RT) and integrate into the genome of the host. Retroviruses typically infect somatic cells. However, retroviruses that are integrated vertically transferred in the following generation when they infect germline cells, resulting in endogenous retroviruses (ERVs). The 1960s saw the discovery of ERVs [17]. Retroviruses have a life cycle that is abruptly split into two separate stages; the stages of infection from cell interaction to viral cDNA integration into the cell genome are referred to as the early phase. In contrast, the expression of viral genes marks the beginning of the late phase, which continues until progeny virions are released and mature. In the early phase, retroviruses must overcome several barriers on their arduous expedition from the surface of the cell to the nucleus. These include the need to navigate the cytoplasm to reach the nucleus, as well as to cross the nuclear and plasma membranes. Additionally, they must avoid or counteract cellular defenses that may obstruct several of these steps. The influx of research on HIV to find novel targets for treatment has improved our knowledge of the retroviral life cycle. Nevertheless, early stages of retrovirus infection remain poorly understood in contrast to subsequent events [18,19]. The particles of the virus adhering to the surface of the intended cell are the first stage of the retroviral replicative cycle. Though it is unclear if specific interactions lead to this binding, it is generally believed that such attachment involves components different from the receptors that initiate the entrance procedure [20]. The entry of HIV in target cells requires receptors like CD4 as well as co-receptors (CXCR4 or CCR5), diverse molecules present on the surface of the cell, like LFA-1, heparan sulfate proteoglycan as well, and nucleolin to allow for the initial adherence of virion to the surface of a cell [21,22]. In HIV-1, mannose present in gp120 identifies DC-SIGN [23]. After binding, retroviral particles engage with the glycoproteins in the viral envelope to enter their target cells by using cell-surface proteins as particular receptors. Many cellular proteins, like GLUT-1 (transporter of glucose) for HTLV and CD4 (T cell surface marker) for HIV, can be used by retroviruses to initiate an infection [24,25].

Viral DNA integration in the genome of the host cell is necessary for the retroviral life cycle for generating the provirus. In order to accomplish this, the reverse-transcribed DNA that is linked to viral proteins and forms pre-integration complexes (PICs) needs to enter the nucleus [26]. PICs from the majority of retroviruses must “wait” for the nuclear membrane to rupture during mitosis since they cannot enter the whole nuclei. It has been demonstrated that the integrase, which has a nuclear localization sequence (NLS), is both required as well as adequate to support the intrinsic build-up of PICs from viruses [27]. Early research on retrovirus integration has shown that parvoviral insertion typically happens in a non-sequence-specific manner, while it might be impacted by nearby chromatin structure [28]. New study areas will certainly be opened by comprehending the specific interactions between cellular and viral counterparts that take place in the initial stages of infection, which can lead to the development of new antiretroviral medications.

Antiviral treatments are becoming considerably more advanced due to improvements in our knowledge of retroviruses and their life cycle. Many antiviral treatments have been created in the last few decades to target different viral components or stages of the viral life cycle, such as genomic replication, fusion, and adsorption [29]. RT and protease inhibitors are examples of antiviral medications that are frequently used to treat HIV. 

As of now, there is neither a vaccine nor a treatment for HIV/AIDS. Combination antiretroviral medication has significantly enhanced treatment; nonetheless, it is not successful in cases where the virus develops resistance, must be taken for a lifetime, and has significant adverse effects. Advances in nanomedicine and nanotechnology in the 21st century are revolutionizing the treatment of viruses that have proven recalcitrant to cure. It has the enormous potential to remarkably improve HIV/AIDS prevention and treatment.

### 2.1. Nanomedicines for Human Immunodeficiency Virus (HIV)

Currently, the most frequent infectious cause of adult mortality worldwide is HIV/AIDS. It has severely harmed social and economic conditions all around the world, especially in emerging nations like those in Sub-Saharan Africa. In nearly 30 years of research, a cure for HIV/AIDS has eluded researchers. Since the US FDA approved the first medication, zidovudine, in 1987, a total of 25 medications have been approved, some of which are accessible in fixed-dose compilation and generic formulations for application in settings with defined resources [30]. However, the development of triple-drug therapy and protease-inhibiting drugs in the 1990s revolutionized AIDS treatment [31]. This ushered in the era of HAART (highly active antiretroviral therapy), involving the simultaneous administration of three or more distinct medication types [32]. 

Nonetheless, substantial adverse effects can occur from some HAART regimens, and daily dosing of one or more medications is required for the duration of treatment. Additionally, some patients become resistant to specific drug combinations, which makes treatment unsuccessful [33]. The fact that, to date, no full cure for AIDS exists highlights the urgent need for ongoing research into novel treatment modalities. Controlled-release delivery systems that could prolong the half-life of antiretroviral medications and maintain them for long periods in blood circulation at therapeutic quantities can greatly improve the scenario. The use of platforms based on nanotechnology for the requisite delivery of these medications has been developed recently along with nanoparticle-based formulations to enhance the medication, decrease the dose frequency that is needed, as well as address patient acquiescence issues [34].

In this regard, a coating of soy lecithin–liposome has been added to gelatin NPs containing stavudine, a nucleoside analogue, to enable dual-functionalized HIV-1 therapy [35]. Stavudine might be liberated gradually in the bloodstream to assist in treating infections in plasma. Still, after being ingested by mononuclear phagocytic cells, the gelatin core could be broken down to liberate the stavudine, which is left over to treat the reservoir of the virus. Solid lipid NPs (SLNs) loaded with the ritonavir (inhibitor of protease) [36] and core graft copolymer (hydrophobic) packed with NNRTI benzophenone–uracil [37] are two recent instances of platforms based on the nano-delivery system for the treatment of HIV. BIC (Bictegravir), an INSTI (integrase strand transfer inhibitor) approved by the FDA, has shown promising results in the treatment of HIV-1 and SIV infections when taken as a single pill regimentation. An effort has been made to increase adherence; researchers have examined the HIV neutralization efficacy of a new protracted nano-formulation of BIC. Polymeric NPs, especially those composed of poly (lactic-co-glycolic acid) (PLGA), are an attractive drug delivery platform because of their biodegradable as well as biologically compatible qualities. HIV-1 preventive and treatment efforts may benefit from the use of BIC nano-formulation as an effective sustained-drug release delivery technology [38]. There have also been numerous reports on combination therapy strategies that make use of nano-delivery technology [39]. Elvitegravir, tenofovir, alafenamide, and emtricitabine, for instance, have all been evaluated as triple combination technology [40]. The subcutaneous administration of the triple therapeutic nanoparticle resulted in undetectable plasma viral loads in animals infected intravaginally with HIV-1. Visceral ribonucleoprotein, or vaults, is an unusual NP-based platform that has been employed for HIV-1 treatment [41]. Since eukaryotic cells naturally contain barrel-shaped particles, they are believed to be extremely biocompatible. The ability to conjugate antiretroviral medications like tenofovir, zidovudine, and elvitegravir on the surface of vault NPs was demonstrated, and the drug–NP coalesce was successful in inhibiting infections caused by HIV-1 in PBMCs (peripheral blood mononuclear cells). 

**Figure 1:**
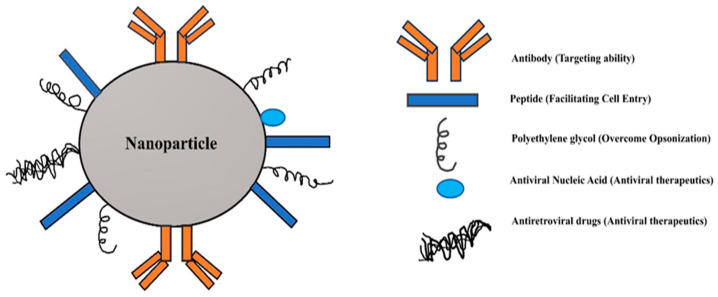
Nanoparticle as anti-HIV therapy.

By the genetic modification of naturally occurring extracellular vesicles with a single chain variable fragment antibody (Ab) component, which has strong binding with gp140 produced on the infected cell surface, nano-formulations are incorporated with supplementary target delivery moiety [42]. The vesicles are injected with curcumin or miR-143 (miRNA-143), a substance that triggers apoptosis, in order to eradicate the particular reservoir of the virus. With HIV-infected cell lines, PBMCs taken from HIV-1-positive patients, and in an animal model with a solid tissue reservoir, the approach demonstrated remarkable efficiency in vitro. Platelet microparticles that deliver lamivudine or tenofovir are different platforms based on biomimetic membrane vesicles which are employed in HIV-1 suppression [43]. Figure 1 depicts NPs as anti-HIV therapy.

Blood–brain barrier (BBB) permeability of individual antiretrovirals presents an important obstacle to antiretroviral therapy (ART), especially in treating viral reservoirs within the central nervous system (CNS). This could lead to neurological manifestations of HIV in the form of HAND or HIV-associated dementia. Researchers have attempted to use cell-based nano-formulations for inhibiting viral proliferation in the nervous system by employing blood-borne macrophages passing through the BBB for ART.

The test was conducted in a mouse model. The ART is made by employing IDV (indinavir) NPs (NP, nanoART) injected into ex vivo-cultivated murine bone marrow macrophages (BMM, IDV-NP-BMM). This study discovered that nanoart can be developed as potential therapeutics for HIV-associated neurological disorders [44].

NPs have the potential to assist in addressing numerous challenges standing in the way of the effective clinical application of RNAi antiviral therapy [45]. Many nanoparticles are used for the delivery of siRNA in cells infected with HIV [46]. For intranasal administration, siRNA targeting Beclin1 has been integrated into cationic PEI (polyethylenimine) nanoplexes, which are biodegradable [47]. Less viral p24 was secreted in the culture supernatant of human astrocytes and microglial cells, indicating that the nanoplexes impeded HIV replication. The nanoplexes were able to penetrate the BBB, reducing the Beclin1 expression level in the brain by 65% when given intraperitoneally to mice in good health. Additionally, ferric–cobalt electromagnetic nanoparticles with beclin1-targeting siRNA have been loaded to treat HIV-1 infections in the CNS [48].

Different formulations of tissue-targeted NPs are created to better manage and prevent HIV infections at specific sites within the tissue. These include formulations for topical or vaginal treatment, along with ones for delivery into the brain for the treatment of neuro-AIDS [49]. Researchers have created hydrogel-core and lipid-shell NPs. MVC (maraviroc) and TDF (tenofovir disoproxil fumarate) were effectively formulated using nanolipogel technology to create ARV-conjugated nanolipogels which are assessed for their physical characteristics and antiviral efficacy against HIV-1 BaL in cell culture. According to the data, hydrophilic small-molecule ARV medication delivery and encapsulation using nanolipogel carriers appears promising. This could lead to an expansion of the nanocarrier system under investigation for HIV prevention [50]. MPLs (multifunctional magneto-plasmonic liposomes) are a composite drug delivery method that combines liposomes with magneto-plasmonic NPs for a ternary-modality approach guided by image, invented by researchers. The antiretroviral medication tenofovir disoproxil fumarate, which is applied for HIV-1 treatment, was enclosed in MPLs that allow for the treatment of the microenvironment of the brain, which is inaccessible to most medications. In MPI (magnetic particle imaging) and MRI (magnetic resonance imaging), the research revealed significant positive and negative contrasts that came from the MPL magnetic center, respectively. X-ray computed tomography (CT) revealed high positive contrast in the gold shell of MPLs. Through magnetic targeting, MPLs were able to achieve increased transmigration across an in vitro model of the BBB.

Additionally, MPLs had the intended therapeutic effects on HIV-infected microglia cells [51]. Using the mannose-targeted poly (propylene imine) dendrimer nanocarrier, efavirenz was administered to human monocytes/macrophages in vitro in a different study [52]. When compared to free medication, the target nanocarrier caused an increase in cellular absorption by 12-fold. Lamivudine was administered utilizing a similar technique, which led to noticeably greater anti-HIV activity for both targeted and nontargeted dendrimer systems as compared to free medicines [53]. In recent work, the medication efavirenz was targeted to macrophages in vitro by conjugating the tetrapeptide tuftsin (Thr-Lys-Pro-Arg) with the same dendrimer [54]. It has been demonstrated that autophagy-inducing peptides, such as Tat-vFLIP-2 or Tat-Beclin1, may eliminate the reservoir of the virus of resting CD4+ T cells in the central memory and macrophages [55]. These particles are coated in lipids. The nanoparticles helped with intracellular transport, and although they caused dose-dependent death in HIV-positive cells, no comparable cytotoxicity was seen in healthy cells. The killing impact was verified in clinically isolated latently infected CD4+ T cells and did not lead to the reestablishment of HIV reservoirs. 

**Figure 2:**
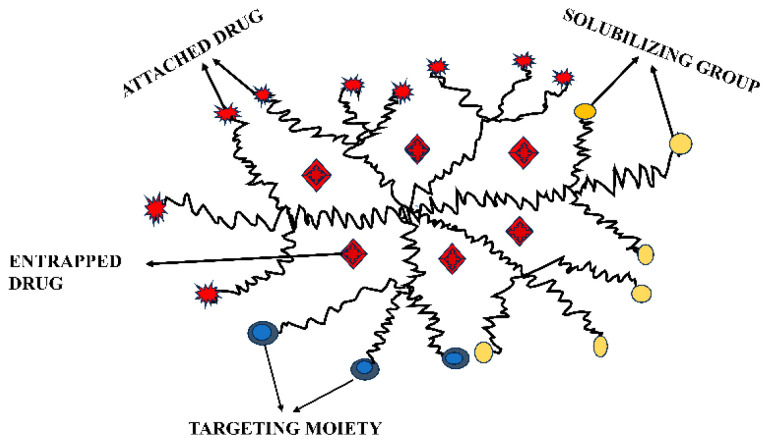
Polymeric dendrimer targeting HIV.

Furthermore, for reactivating the cytotoxic T cell pool, lipid-coated PLGA nanoparticles laden with different agents that can reverse latency, like JQ1 or ingenol-3-angelate, have been chemically attached with an anti-CD4 monoclonal Ab via maleimide–thiol click chemistry [56]. The CD4+ T cells were vulnerable to being destroyed by additional antiviral treatments when they were reactivated. When injected subcutaneously into mice, the anti-CD4-functionalized NPs demonstrated a more than two-fold selective deposition in lymphatic cells as compared to the nontargeted formulation. Figure 2 depicts a polymeric dendrimer targeting HIV. 

Lactoferrin (Lf) shares a structure with transferrin and is secreted by ectodermal tissue. Lf can be applied to drug nanocarrier functionalization. Additionally, one study has demonstrated that antiviral medications are encapsulated in Lf nanoparticles. Through sol–oil chemistry, zidovudine nano-encapsulation into Lf nanoparticles has been accomplished. Although zidovudine has an excellent 50–75% bioavailability and is an effective antiviral medication, it can also cause organ damage, neutropenia, and bone marrow suppression. The produced nanoparticles in the study had a size of 50–60 nm, a drug encapsulation effectiveness of 67%, and good physical stability at both ambient temperature and 4 °C [57]. Moreover, neither the drug content nor the particle size significantly changed. When lipophilic nanoparticles loaded with efavirenz were taken orally, the anti-HIV-1 effects were equivalent to those of the medication in its free form. Furthermore, drug-loaded nanoparticles had better pharmacokinetic properties and decreased organ toxicity when compared to free efavirenz, suggesting that this nanoformulation is a secure nano platform that can improve drug delivery [58].

### 2.2. Nanomedicines for Influenza Virus

Influenza viruses are significant respiratory tract infections in humans that cause seasonal epidemics and sometimes global pandemics [59]. Currently, there are only two medications approved by the U.S. FDA to combat various influenza A strains and subtypes: blockers of the matrix-2 (M2) protein ion channel, like rimantadine and amantadine, and inhibitors of neuraminidase, such as zanamivir and oseltamivir [60]. In recent years, drug-resistant strains have evolved, which is a prime reason for concern. Therefore, alternative therapies are required to treat such drug-resistant strains, and nanotechnology plays a crucial role. Antiviral medications are frequently delivered via metal-based NPs to treat influenza infections [61]. Amantadine, a nicotinic antagonist, and a noncompetitive N-methyl-D-aspartate antagonist have been linked with silver nanoparticles (AgNPs) to develop a treatment for influenza that targets NA inhibitors. These agents can impede the replication of influenza viruses. Because the antiviral drug-conjugated nanoparticles prevented intracellular ROS production and caspase 3-mediated apoptosis, they can stop the invasion of influenza and preserve cells [62]. SeNPs, which are selenium nanoparticles, have been modified with oseltamivir, zanamivir, and amantadine in a study conducted by Xia and colleagues in 2018. In addition, to treat influenza infections, SeNPs were also used to deliver umifenovir, an entry inhibitor, and ribavirin, a nucleoside analogue. Another often used platform is polymeric nanoparticles, which have been used to specifically transport favipiravir and miR-323a to influenza viruses using a sialic acid target moiety [63]. Similarly, two strong vacuolar ATPase inhibitors, diphyllin and bafilomycin, were successfully loaded into polyethylene glycol (PEG)-functionalized polylactic acid NPs (PLGA nanoparticles), as well as showed reduced toxicity to cells and increased antiviral efficacy by two and five times, respectively [64]. Research is conducted to investigate the effectiveness of zinc oxide NPs (ZnO-NPs) and PEGylated zinc oxide NPs in combating the H1N1 virus strain. The findings of this study indicate that PEGylated ZnO-NPs could serve as an innovative, potent, and encouraging treatment for H1N1 influenza virus infection. Subsequent research will investigate the precise antiviral mechanism of these nanoparticles [65]. DNA fragments have been used to functionalize titanium dioxide (TiO_2_) NPs, which have been linked with polylysine to target the 3′ non-coding regions of influenza A virus. This new type of nanocomposite has been found to effectively combat the influenza A virus, and it also has the ability to enter cells without requiring transfection agents [61]. The administration of RNAi treatment via nanoparticulate delivery has been widely used to treat influenza infections. In this regard, gene silencing and preventing influenza viral replication have been achieved by the use of calcium phosphate NPs [66] and chitosan NPs [67]. Titanium oxide nanocomposites are utilized to deliver deoxy ribozymes, which are DNA enzymes capable of cleaving viral RNA. In vitro studies have demonstrated that the use of deoxy ribozymes in reducing H5N1 viral titers could achieve a reduction of approximately 2650-fold. Remarkably, siRNAs have been delivered by silica-coated microcapsules following sol–gel production [68]. The release of siRNA cargo in the cytoplasm could be facilitated by the microcapsules being endocytosed into cells and breaking down. The engineered siRNAs can effectively target the viral nucleoprotein (NP) gene and inhibit the expression of viral proteins by downregulating the target mRNA once they enter the cytosol. The siRNA microcapsules successfully prevented H1N1 replication and were an improvement over transfection using the conventional siRNA/PEI polyplexes. Peptides that can penetrate cells have been used to increase the intracellular distribution of siRNAs since they require entry into the cytosol to be effective [69].

### 2.3. Nanomedicines for Human T-Lymphotropic Virus

HTLV is an oncovirus family of human retrovirus, well recognized for inducing immune-suppressive and inflammatory diseases. HTLV-1 is the most clinically relevant member of this family and the first infection that has been demonstrated to cause cancer. Nowadays, most people agree that HTLV-1 is among the most prolific human oncogenes [70]. Although about 95% of people infected with HTLV are asymptomatic, the remaining 5% can show malignancy, opportunistic infections, or inflammatory diseases. HIV-1 and HTLV-1 have similar cellular tropism and modes of propagation. HIV-1/HTLV-1 concurrent infection has been studied often, referring to the co-occurrence of these viruses in many regions of the world. According to one of the earliest studies describing co-infection, roughly 7% of people with AIDS or an illness associated with AIDS are also chronic for HTLV-1 [71]. Clinical trials are performed to enhance the efficiency of treatment. Relatively poor results were obtained with chemotherapy (first-generation), which included CHOP (cyclophosphamide, vincristine, doxorubicin, and prednisone) as well as CHOP-like regimens. Clinical trials of regimens based on the Lymphoma Study Group (LSG15) have demonstrated greater efficacy nearly two decades later. A minimum of three drugs are included in LSG15-based regimens: VECP (vindesine, etoposide, carboplatin, and prednisone) and VCAP (vincristine, cyclophosphamide, doxorubicin, and prednisone) [72]. Instead of eliminating HTLV-1-infected cells, the majority of these treatments aim to reduce clinical symptoms. As a result, research is currently focused on medications that may alter the anti-HTLV-1 immune response and lower the proviral load of HTLV-1. Currently, several novel medications or combination therapies for the treatment of ATL are being researched. Anti-metabolites like Cladribine, Clofarabine, and Pralatrexate are under trial. Cladribine is a purine nucleoside analogue which cannot be metabolized by adenosine deaminase. Lymphocytes that possess strong deoxycytidine kinase activity and phosphorylated derivatives of cladribine accumulate, leading to DNA strand breakage and cell death [73]. Cladribine is unique, such that it damages quiescent and rapidly dividing cells. A metabolic inhibitor of folate analogues called pralatrexate can competitively inhibit polyglutamylation by folylpolyglutamyl synthetase, an enzyme, and competitively inhibits dihydrofolate reductase. This depletes thymidine and other biologic substances [74]. The nanoformulation of these drugs can significantly improve the treatment of HTLV infections. Combining nanoparticles for vaccine formulation can stop HTLV-I from spreading. As a delivery mechanism or adjuvant, polymeric NPs like CHT (chitosan) or TMC (trimethylchitosan) might enhance immune responses to peptide antigens. Both NPs have shown potent immune-adjuvant potential and can be used to develop an effective vaccine against HTLV [75].

### 2.4. Nanomedicines for Hepatitis C Virus

The most prevalent blood-borne viral infection, hepatitis C, is largely caused by the hepatitis C virus (HCV) and affects the liver. The inability of the body to eliminate the virus is responsible for approximately 80% of acute HCV infections progressing to chronic infections [76]. Interferons were the first drug used in HCV therapy, followed by direct-acting antivirals (DAAs) and host-targeted antivirals (HTAs). Even with these advancements in treatment, many people worldwide still lack access to secure and inexpensive anti-HCV medication. Current research efforts are directed towards creating affordable and efficient therapies to guarantee fair accessibility to HCV care. Significant efforts are focused on nanoparticles (NPs) for the delivery of various regimens, encompassing anti-HCV vaccines and diagnostic and therapeutic agents [76]. These drugs were tethered to or enclosed within NPs with varying compositions, including metallic, lipid, and polymeric NPs [76]. The stability of anti-HCV medications has faced numerous obstacles in their attempts to transport them to their intended locations safely. NPs were designed with specific features in mind to address these difficulties, such as maintaining the medication’s impact while reducing the frequency of administration and, in turn, minimizing any potential negative effects. Anti-HCV medications have a sustained impact because of the PEGylation of NPs, drug diffusion control from the NP matrix [77], and electrostatic interactions with carriers that are oppositely charged [78].

Furthermore, nanotechnology can be used to easily protect anti-HCV medicines and increase serum stability [78]. Additionally, nanotechnology can lower the cytotoxicity of some of these therapeutics, like ribavirin (RBV), when coupled with nanoparticle-based delivery, as demonstrated by the decreased hemolytic anemia with NPs [79]. The most important feature of NPs in HCV therapy is their targeted delivery to the liver, which means that anti-HCV medications could be delivered to hepatocytes, which is the primary site of HCV replication. Anti-HCV medications have been known to target the liver by the surface decorating of NPs with particular moieties such as galactose (Gal) [79], hyaluronic acid (HA), or vitamin E (VE) [80].

**Figure 3:**
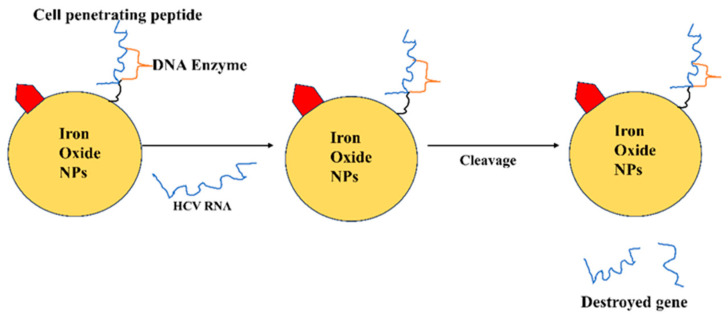
Iron oxide NPs with DNAzyme used to target the HCV replication.

Iron oxide NPs can target hepatocyte receptors like asialoglycoprotein and HA receptors for delivery to hepatocytes. Since it is extremely difficult to find new medications for the treatment of HCV, ongoing research is being conducted to reduce the off-target effects and toxicities associated with the medications that are currently on the market, as well as to enhance delivery methods using emerging technologies like nanotechnology. For this method, several kinds of lipid, metallic, and polymeric NPs were used and investigated. Several NPs with HA and HA decoration were created with the express purpose of targeting liver cells [81]. When HCV infects hepatocytes, RBV (ribonucleoside analogue) exhibits antiviral action. On the other hand, it heavily accumulates in red blood cells (RBCs), which leads to hemolytic anemia when used frequently [82]. Modifying RBV dispersion by nanotechnology has been thoroughly researched in order to reduce RBC absorption. In polymeric NPs made of poly (D, L-lactic acid; PLA) as well as arabinogalactanpoly (L-lysine; AG-PLL), RBV monophosphate (RMP, prodrug) was steadily loaded. Without causing a large amount of drug release into the bloodstream or accumulation of RBV in erythrocytes, the carrier was able to precisely deliver the medication to the liver. The polymer concentration of arabinogalactan (AG), a polysaccharide with Gal side chains, was the cause of liver targeting. Hepatocytes’ asialoglycoprotein receptor is specifically bound by gal-decorated NPs, causing endocytosis and the release of cargo from inside the liver cells [83]. Figure 3 depicts iron oxide NPs with DNAzyme to target the HCV replication.

**Figure 4:**
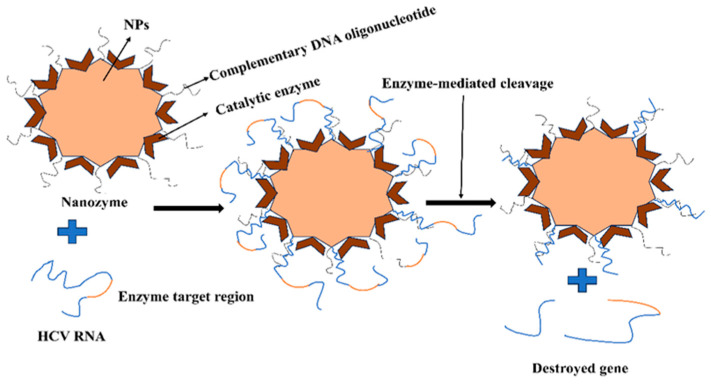
Functionalized nano enzyme used to target hepatitis C virus.

Researchers have also created Gal-decorated NPs to deliver RBV to the liver with little cytotoxicity, all without the need for PLL [84]. The interaction between PLA and the poly (L-glutamic acid)/AG [PLG–AG] conjugate produced these NPs. There have been reports of other polymeric NPs based on poly (glycerol adipate) (PGA) as an RBV delivery system that targets the liver and reduces RBC buildup. According to this study, NP absorption was substantially greater in HCV-infected cells than in nonviral-infected cells. Additionally, galactosylated-α,β-poly(N-2-hydroxyethyl){[(2-aminoethyl carbamate)] was loaded with the tripalmitate prodrug of RBV. The prodrug’s entrapment efficiency is enhanced by the -co-[2-(1,2-dipalmitoylsn-glycero-3-phosphoethanolamine-N-succinyl)-amidoethylcarbamate]}-D,L-aspartame (PHEA-EDA-DPPEGAL) copolymer because of its high hydrophobicity [84]. Researchers have created a customized nanozyme (48 ± 2 nm, 14 ± 1 mV) with AuNPs as the core and two neighboring components as the shell: endoribonuclease (RNase A), which is complementary to the target HCV RNA at 322–339 nucleotides, and DNA oligonucleotide, which can cut HCV RNA [85]. A test using nanozyme RNase activity revealed that the enzymes worked along with oligonucleotides to specifically target and cleave HCV RNA into two pieces according to particular sequence and location requirements, resulting in a 99.9% reduction in HCV RNA levels. Consequently, these intracellular nanomachines are powerful treatments against HCV through significant cooperative mechanisms, together with strong target stability and safety. With a superior safety profile and patient compliance than interferon-based regimens, DAAs were able to completely transform the treatment of HCV. When using DAAs, a few mild adverse medication responses, such as headache, gastrointestinal problems, and exhaustion, have been documented [86,87]. Widespread use of these medicines against various genotypes has been approved by the FDA [86]. Figure 4 illustrates a functionalized nano enzyme used to target the hepatitis C virus.

The two main issues with these DAAs are their cost and the potential for resistance to develop as a result of viral alterations. There have been no reports of DAAs as nanomedicines yet. Researchers ought to be encouraged to investigate the various advantages that could be applied to DAAs through the use of nanotechnology.

### 2.5. Nanomedicines for SARS-CoV-2

Public health is particularly worried about virus-related diseases, and the latest SARS-CoV-2 pandemic shows once more how dangerous viral infections are to life as we know it [88]. SARS-CoV-2 caused a pandemic in December 2019. This resulted in a public health disaster. Scientists all around the globe investigated the creation, manufacturing, and standardization of SARS-CoV-2 quick diagnostic tests and therapy due to the COVID-19 pandemic’s consequences on the world economy, public health, and shortages in medical and diagnostic infrastructure [89]. Together with its physicochemical characteristics, nanomedicine may offer a treatment strategy that can help end the conflict between CoVs and host cells. NPs that have viral antigens or antibodies covering them could be applied to SARS-CoV-2 and any resurgence of CoV. Organic NPs have been used to supply targeted antivirals, such as acyclovir, zidovudine, dapivirine, and efavirenz, as well as to improve drug bioavailability and boost effective drug delivery action [90]. The main disadvantage of antivirals is that they do not target specifically, which results in host cell cytotoxicity that organic NPs can counter. Antimicrobial medication nanoencapsulation may lead to the development of safer COVID-19 and other viral illness treatments [91]. Magnetic nanoparticles (MNPs), which have emerged as an encouraging thermodynamic tool in biomedical applications involving drug administration, diagnostic imaging, and innovative therapies, are a result of the rapid advancements in nanomedicine. Extensive preclinical and clinical research has demonstrated their ability to target delivery, regulate drug release, functionalize, and follow an image. Through active or passive targeting, MNPs can concentrate at the intended site where they can functionalize with targeting ligands and medications to increase therapeutic efficacy [92,93].

Dexamethasone is an anti-inflammatory steroid. According to reports, dexamethasone medication reduced the number of patient deaths linked to COVID-19 by 35%. The NFs of dexamethasone were postulated based on the widely held belief that NPs aggregate potently in macrophages upon intravenous injection and inhalation. A few persistent, potentially fatal SARS-CoV-2 symptoms include the emergence of cytokine storm, oedema, and fibrosis. Three different methods that dexamethasone nanomedicines may aid in the daily management of COVID-19 disease have been proposed [94]. Firstly, by targeting inflammation, initiating phagocytic cells of myeloid, lungs, blood, and the lymphatic system. Secondly, the anti-fibrotic action potential of the nanomedicine dexamethasone formulation will be improved to satisfy an urgent medical need to manage COVID-19. One very effective anti-edema medication is dexamethasone. Lastly, nano-formulating can further enhance this impact by gradually boosting medication availability and drug action in hyper-activated immune cell populations in the inflammatory portions of the lung.

In order to mitigate cytokine storms and their associated pathological effects, macrophage-targeted nanomedicines may be utilized to improve patient outcomes in cases of severe COVID-19 infections [95]. The origin and structure of the NPs used in macrophage-targeting approaches can vary greatly. Still, there are two main pathways through which macrophage uptake usually happens: non-specific phagocytosis, which is passive targeting controlled by the physical properties of NPs, or endocytosis, which is active targeting mediated by receptors [96]. These NPs mainly aggravate inflammatory or infected areas. Different techniques can be used to design natural or synthetic NPs such that they can more easily target macrophages.

Gold nanoparticles can be employed in vaccinations, colorimetric-based viral detection assays, and as virucidal agents to strengthen mucosal cellular immunity. Titanium can operate both as a virustatic and virucidal agent. Because of their virucidal characteristics, silver nanoparticles stop the virus from infecting the host cell. The nanoparticles of iron oxide are employed in SARS-CoV-2 vaccines, have antiviral qualities, and are applied for quick viral elimination and detection. Silica nanoparticles are used as a preventive measure against viral entry into target cells, as well as an adjuvant vaccination. In addition, silica can be employed in sandwich immunoassay detection based on fluorescence [97].

## 3. NanoVaccines

Recently, NPs have shown great promise in the field of medicine due to their unique physiochemical properties, tiny size, and improved surface-to-volume ratio, which facilitates the absorption, conjugation, and encapsulation of molecules for target delivery [98]. The creation of vaccinations is essential to the successful management of a number of deadly illnesses. Nevertheless, effective preventative and therapeutic vaccinations are still needed to fully eradicate fatal illnesses, including cancer, malaria, HIV, and severe microbial infections. As a result, proper vaccination candidates must be created to trigger the right kind of immune response. It has been discovered that nanotechnology plays a special role in vaccine design, giving them increased efficacy and specificity. Over the past ten years, there has been a lot of interest in using nanoscale materials like virus-like particles, liposomes, polymeric NPs, and protein NPs as possible delivery vehicles for vaccine antigens and adjuvants [99]. These materials offer several advantages, including enhanced antigen stability, targeted delivery, and prolonged release, because the antigens and adjuvants are either encapsulated within or decorated on the surface of the NP. The numerous difficulties faced in the creation of vaccines can be addressed by engineering immune responses through the flexibility of nanomedicine design [100]. In order to create viral-mimicking NPs, we must act and think like viruses and make use of the many alluring qualities that they have to offer. We can employ a variety of variables, such as age susceptibility, the environment, and the affinities of individual viruses to particular cells, tissues, and organs, to accomplish targeted and selective delivery. For these reasons, using viral shells as nanocarriers for the creation of nano vaccines may be the best option. The genetic component within the virus’s shell is the most toxic and dangerous part. However, by studying the various viral shells that have coexisted with humans for years and creating synthetic structures that mimic them, we can create a variety of nanocarriers that have distinct viral-mimicking qualities [99]. Viral characteristics such as immune system evasion, physiochemical traits, biodistribution, tissue tropism, specialized high-affinity receptors, cell entrance, and endosomal escape make them interesting candidates for application in the development of nano drugs and nano vaccines [100].

In the nearly three decades following the discovery of HIV/AIDS, the hunt for a secure and reliable vaccine has been difficult. Lipid-based methods have been extensively studied for delivering HIV/AIDS vaccines. A previous study [101] showed that the HIV gp160 protein encapsulated in a liposome and administered nasally to mice produced high titers of neutralizing antibodies specific to gp160. The liposomes were created by combining cholesterol, phosphatidylethanolamine, sphingomyelin, phosphatidylserine, and phosphatidylcholine. Additionally, the HIV gp41 protein was given using a range of liposome sizes (110–400 nm), which caused mice and rabbits to develop robust antibody responses. Since there are currently no vaccines that may cause an antibody response to HIV that is broadly neutralizing, it seems unlikely that an antibody response to a monovalent encapsulated antigen will be sufficient for an HIV vaccine that sterilizes [102]. Therefore, it is necessary to develop these nano-delivery systems to incorporate a range of HIV epitopes, possibly even ones that are designed to promote antibody response production and access to regions of the HIV glycoproteins that are not evoked by acute HIV infection. The use of NPs as a delivery mechanism, however, in this work decreased the amount of HIV antigen and flagellin (adjuvant) required to produce a cellular response (proliferation and IFN-γ production) that is comparable to that seen with Freund’s adjuvant [103]. With ICMVs, the benefits of HIV Env trimer nanoencapsulation over soluble antigen have also been shown.

Trans-mucosal immunization remarkably produces very strong protective mucosal immune responses. For instance, because chitosan not only promotes mucosal vaccine delivery but also significantly increases antigen-specific IgG and IgA antibody responses, it has been suggested as a penetration enhancer to increase the trans-mucosal bioavailability of the HIV envelope glycoprotein (CN54gp140) vaccine. HIV vaccine’s permeability across mucosal barriers can be significantly increased by using nanotherapeutic approaches [104]. Recently, epidermal Langerhans cells (LCs) were the target of a study that used DNA and a polymer to create artificial “pathogen-like” nanoparticles in the 50–24 nm range. In order to transport targeted DNA to LCs, they effectively encapsulated the DNA using polyethyleneimine (PEI) that was altered by covalently bonded mannobiose molecules. Following topical administration of the nanoparticles, the human participants exhibited strong immunological responses, as the nanoparticles effectively loaded the vaccine into the LCs to travel into lymph nodes [105].

It is ideal to have complete protection in the mucosal respiratory tract against the influenza virus. Thus, research into NP vaccinations against the influenza virus is carried out. A combination of VLPs and TIV used to immunize mice is one of the most basic NP vaccines tested against the influenza virus. Based on IgG, IgG2a, and IgA titers in blood and bronchoalveolar lavage samples, the VLP/TIV combination was found to be more effective than free TIV for inducing anti-influenza immunity, particularly following intranasal immunization. Additionally, rabbits immunized with influenza whole virus (WV) encapsulated into the mucoadhesive carrier chitosan were able to successfully immunize their nasal mucosa with NPs [106,107]. In mice, an NP vaccine against H5N2 was generated by encapsulating the trimer of H5 hemagglutinin in polyanhydride NCs. This approach resulted in a strong TCD4+ response and large titers of neutralizing antibodies. Additionally, animals were found to be considerably protected following an H5N2 nasal challenge [108].

In order to stop HTLV-I from spreading, a reliable vaccine is needed. As a delivery mechanism or adjuvant, polymer-based NPs such as chitosan (CHT) and trimethyl chitosan (TMC) might enhance immune responses to peptide antigens. Recombinant proteins (env23 and env13) of gp46 were loaded into CHT and TMC NPs using the direct coating of antigens with positively charged polymers. Each antigen-loaded CHT and TMC NP measured approximately 400 nm in size. For four weeks, the physical stability of NPs was monitored. For roughly fifteen days, both formulations proved to be stable. Following the nasal and subcutaneous immunization of mice, the immunogenicity of NPs loaded with antigens was investigated. At intervals of two weeks, three immunizations totaling 7.5 µg of antigen were administered. Both of them have shown better immunoadjuvant properties. Env23 has better potential as an antigen for an effective HTLV vaccine [75].

The membrane-anchored SARS-CoV-2 full-length spike (S) protein is encoded by the mRNA-based vaccine BNT162b2, which is encased in lipid NPs and has two proline mutations changed to prevent S in a prefusion conformation [109]. Furthermore, the mRNA exhibits an N1-methyl-pseudouridine base alteration for uridines in order to improve protein expression and suppress the innate immune response [110]. After the initial two intramuscular doses of thirty micrograms of BNT162b2 mRNA, the antibody titers start to decline a few months after the final immunization; hence, a booster dose is required. Research has demonstrated that chitosan and its derivatives possess potential anti-SARS-CoV-2 action, operate as useful adjuvants in vaccines, and exhibit direct antiviral activity [111]. For instance, plasmid DNA encoding the nucleocapsid SARS-CoV-2 was delivered intranasally using chitosan nanoparticles as a stimulant. This causes the SARS-CoV-2 spike protein to be secreted, which, in a rat model, can compete with the live coronavirus for binding to human ACE2 receptors [112].

## 4. Nanoherbals

Although there are many medicinal plants available worldwide, it is yet unknown how to use plant components or secondary metabolites to treat severe retroviral diseases like HIV. Numerous secondary metabolites found in plants possess the ability to combat this virus and other opportunistic viruses. The use of allopathic medications causes mutations in the human body, and the excessive cost worries both the medical community and the patients. An inexpensive, side-effect-free alternative nanomedicine for retroviral illness is a medicinal plant using nanotechnology. Plants were essential to human life in the ancient era. Our forefathers started to recognize the uses of plants for food, medicine, and leisure as they advanced in civilization. They created their medical system by repeatedly trying and failing to determine the right application, dosage, and procedure for therapeutic herbs. Alkaloids and flavonoids, two types of secondary metabolites found in plants, may have the ability to combat viruses and manage viral illnesses [113]. Because traditional medical systems are less expensive and have fewer side effects than Western medicine, they are becoming more and more popular as an alternative. For instance, 40% of people in China, 71% of people in Chile, 40% of people in Colombia, and approximately 65% of people in India rely on traditional medicine for their basic requirements. Their use in the treatment of a few serious illnesses, such as HIV/AIDS, has also been discussed. However, the absence of scientific evidence and analysis renders it unacceptable in today’s society. Therefore, in order to separate the active components from medicinal plants, it is crucial to perform phytochemical, in vitro, and in vivo analysis and fractionation. The only treatment option for this viral illness other than pharmaceuticals may be the use of medicinal herbs.

Rubia cordifolia, commonly referred to as Indian madder or common madder, is a type of flowering plant in the Rubiaceae family that has been shown to have anti-HIV properties. Quinolizidine, alkaloids, lectins, non-protein amino acids, tannins, and alkylsterols are found in R. Cordifolia [113]. By reducing the expression of the LTR gene and the synthesis of the p24 antigen, the extract demonstrated the possible suppression of HIV. To test against HIV in vitro, several plant parts, including leaves, stems, roots, and entire plant extracts, were produced using isopropyl alcohol and water. There was encouraging anti-HIV activity found in the extracts. In traditional medicine, Rhizophora lamarckii, also known as Red Mangrove, has been widely used to treat leprosy, asthma, backaches, fever, lesions, sore throats, jaundice, and lesions. Stable nanoparticles with a size of 12–28 nm were created by the aqueous plant extracts that were employed to decrease the silver ions to nano size. The reverse transcriptase enzyme was inhibited by the nanoparticles, demonstrating potent anti-HIV-1 action even at low doses. The binding of nanoparticles greatly enhanced the plant extract’s anti-HIV efficacy [114]. Numerous antiviral traits of the Rhizophora species, including those against HIV and hepatitis B virus, have already been demonstrated [115].

Silver NPs (AgNPs) are the subject of most investigation because they have been known to be used for centuries to treat burns and wounds in traditional medicine. The scientific evidence supporting the antibacterial activity of silver nanoparticles has been investigated and verified by numerous researchers [116]. In one such study, Alysicarpus monilifer leaf extract was used to synthesize AgNPs, which showed promise in combating two common infections in HIV-positive individuals: coagulase-negative staphylococci (CoNS) and methicillin-resistant Staphylococcus aureus (MRSA), types of bacteria that primarily cause opportunistic infections related to tuberculosis.

Combining natural remedies with pharmaceutical nanotechnology can result in positive and promising therapeutic outcomes. When plant extracts and poorly soluble phytoconstituents are delivered by nanoformulations, improved clinical and therapeutic outcomes may result [117]. Phytoconstituents with antiviral potential have been delivered via a variety of delivery systems, including hydrogels, phytosomes, microspheres, transferases, self-nano emulsifying drug delivery systems (SNEDDSs), etc. Numerous effects were demonstrated by these nanoformulations, including higher therapeutic action, delayed metabolism, improved oral solubility, and systemic bioavailability. In one instance, intestinal absorption was accelerated by chitosan nanoparticles containing EGCG and catechin [118]. Myricetin is a naturally occurring flavonoid that was found to have a much higher solubility profile when it was placed into a polymeric nanoparticle carrier [119]. The red blood cell encapsulation of flavonoids can increase their bioavailability and antiviral activity. The erythrocyte membrane’s oxidative damage was lessened by the flavonoids [120]. According to a number of studies, RBCs are essential for the transportation and accessibility of quercetin in the bloodstream [121]. It is surprising to discover how flavonoids in chitosan particles still have antioxidant properties and can be employed to fight circulatory free radicals [122]. Quercetin was successfully encapsulated using the most uniformly distributed kind of polylactic acid-4 nanoparticle, allowing for the delayed release of quercetin [123]. Researchers increased Apigenin’s oral bioavailability by adding apigenin to the water-in-oil emulsion technique [124]. Baicalein’s low permeability and solubility led researchers to employ a micellar composition containing sodium taurocholate and Pluronic P123 copolymer as carriers, which greatly enhanced baicalein’s oral absorption [125]. By adding oleanolic acid to SMEEDS, its systemic bioavailability was increased [126]. PLGA was employed to create andrographolide-loaded microspheres in order to get around these restrictions [127]. Studies have shown that silver nanoparticles (AgNPs) were synthesized using methanolic extracts of ginger (Zingiber officinale) and strawberries (Fragaria ananassa Duch.) in order to test their SARS-CoV-2 inhibitory potential [128]. The development of nanotechnology for Indonesian Jamun to combat SARS-CoV-2 is the subject of several investigations [129].

East Asians have traditionally included the edible algal fucoidan in their diets. The polymer has been found to possess a number of medicinal properties recently, including antibacterial, antithrombotic, antiviral, anti-inflammatory, and anti-cancer properties. It was possible to create fucoidan nanoparticles with effective drug loading and release. Additionally, the polymer has been utilized to coat several inorganic and organic NPs, giving these systems biocompatibility properties. Researchers reported using cyanoxyl-mediated free radical polymerization to prepare fucoidan mimic glycopolymer. When tested against the herpes virus, the synthesized polymer exhibited antiviral activity that was analogous to those found in natural polymers [130]. The rhizomes of the plant Curcuma longa contain curcumin, which has anti-inflammatory, anti-apoptotic, and antioxidant properties that have biological effects. Curcumin can regulate both viral replication and inflammation in influenza infections, according to some of the most important findings about the drug. Research indicates that curcumin’s antiviral and anti-inflammatory properties could offer therapeutic benefits in managing harmful inflammation in COVID-19 patients [131]. In an outpatient context, the oral nanoformulation of curcumin can dramatically shorten the recovery period for individuals with mild to moderate COVID-19 [132].

## 5. Conclusions

Nanotechnology can have a measurable impact on multiple viral diseases, including HIV/AIDS therapy and prevention. Antiretroviral medication delivery by nanotechnology platforms may improve treatment choices. The efficacy of treatment may be enhanced by better patient adherence to medication schedules fostered by a controlled and prolonged release of the medications. Targeted nanoparticles against specific receptors have been used for delivery to specific cell types like macrophages, which are important HIV viral reservoirs, using ligands such as mannose, galactose, tuftsin, and fMLF peptides. In the future, it may be possible to enhance the treatment of viral reservoirs by delivering two or more antiviral medications using a nanoparticle system. Nanomaterials can prevent viral replication and also act as carriers for antiviral drugs. Some inorganic nanoparticles, such as silver and fullerene dendrimers, can improve the antiviral properties of other molecules, like gold nanoparticles, or have antiviral effects themselves. Nanotechnology has the potential to improve newer therapeutic methods like gene therapy and immunotherapy. One of the most active areas of nanotechnology research is the nonviral delivery of siRNA. Early research indicates that vaccinations against HIV/AIDS based on nanotechnology may also be promising. NPs are an excellent substitute for viral vectors because of their capacity to selectively target particular cells and release antigens in a regulated and prolonged manner. In animal investigations, it has been demonstrated that lipid- and polymer-based nanoparticles can trigger HIV-specific antibodies and cellular immune responses. In the future, the only source of alternative medicine for the treatment of HIV and other opportunistic viral illnesses may be medicinal plants’ secondary metabolites combined with NPs or nanomedicine. More studies are required, coupled with validated proof employing single herb or multi-herb formulations for managing HIV viral illness, as there has only been a small amount of research conducted so far utilizing medicinal plants and nanoparticles.

The current pandemic caused by COVID-19 highlights the fact that despite the significant technological advancements of the past century, modern medicine is not adequately equipped to handle the emergence of new viral infections. As researchers continue to work tirelessly to develop a vaccine to halt the spread of SARS-CoV-2, nanotherapeutics can serve as a temporary measure to slow down the progress of the virus. Treatments for coronavirus-related infections may involve the use of virucidal nanomaterials and nanodecoys [133]. Silver-based nanomaterials such as AgNPs, Ag nanowires, and Ag colloids have been proven to hinder porcine-transmissible gastroenteritis coronavirus infections in swine testicular cells [134]. Different kinds of nanomaterials have been demonstrated to suppress Middle East respiratory syndrome coronavirus (MERS-CoV) and FcoV, respectively. These include GO–AgNP conjugates [135] and polyanionic dendrimers [136]. Graphene’s antiviral properties have recently been applied practically, whereby surgical masks are coated directly with the material to achieve self-sterilization [137]. When it comes to clinical translation, antiviral nanotherapeutics face a number of significant obstacles that must be taken into account. Even though a large number of nano drugs have been licensed for use in human patients, any new nanoscale platform technology must have its safety profile thoroughly assessed. Many benefits come with using drug vectors to release bioactive natural compounds in a controlled manner through a range of nanoformulations: they are non-toxic, biodegradable, able to incorporate both hydrophilic and lipophilic chemical compounds, have a longer half-life in circulation, can target specific organs or tissues, lessen the toxicity of the active ingredient, and frequently increase its bioavailability [138]. The antiviral activities of nanomaterials and nanodecoys, in particular, need to be demonstrated in the context of minimal toxicity. Considering the above literature review, it is very clear that nanotechnology-based nanomedicines will certainly have great promise and overcome the existing challenges of drug delivery.

## References

[1] Fonkwo P.N. (2008). Pricing Infectious Disease: The Economic and Health Implications of Infectious Diseases. EMBO Rep..

[2] Irwin K.K., Renzette N., Kowalik T.F., Jensen J.D. (2016). Antiviral Drug Resistance as an Adaptive Process. Virus Evol..

[3] Ulmer J.B., Valley U., Rappuoli R. (2006). Vaccine Manufacturing: Challenges and Solutions. Nat. Biotechnol..

[4] Gao F., Weaver E.A., Lu Z., Li Y., Liao H.-X., Ma B., Alam S.M., Scearce R.M., Sutherland L.L., Yu J.-S. (2005). Antigenicity and Immunogenicity of a Synthetic Human Immunodeficiency Virus Type 1 Group M Consensus Envelope Glycoprotein. J. Virol..

[5] Li G., De Clercq E. (2016). HIV Genome-Wide Protein Associations: A Review of 30 Years of Research. Microbiol. Mol. Biol. Rev..

[6] Rudometov A.P., Chikaev A.N., Rudometova N.B., Antonets D.V., Lomzov A.A., Kaplina O.N., Ilyichev A.A., Karpenko L.I. (2019). Artificial Anti-HIV-1 Immunogen Comprising Epitopes of Broadly Neutralizing Antibodies 2F5, 10E8, and a Peptide Mimic of VRC01 Discontinuous Epitope. Vaccines.

[7] Rathore U., Purwar M., Vignesh V.S., Das R., Kumar A.A., Bhattacharyya S., Arendt H., DeStefano J., Wilson A., Parks C. (2018). Bacterially Expressed HIV-1 Gp120 Outer-Domain Fragment Immunogens with Improved Stability and Affinity for CD4-Binding Site Neutralizing Antibodies. J. Biol. Chem..

[8] Korber B., Gaschen B., Yusim K., Thakallapally R., Kesmir C., Detours V. (2001). Evolutionary and Immunological Implications of Contemporary HIV-1 Variation. Br. Med. Bull..

[9] Powell R.L., Urbanski M.M., Burda S., Kinge T., Nyambi P.N. (2009). High Frequency of HIV-1 Dual Infections among HIV-Positive Individuals in Cameroon, West Central Africa. JAIDS J. Acquir. Immune Defic. Syndr..

[10] Barouch D.H. (2008). Challenges in the Development of an HIV-1 Vaccine. Nature.

[11] Gates B. (2018). Innovation for Pandemics. N. Engl. J. Med..

[12] Tamori A., Enomoto M., Kawada N. (2016). Recent Advances in Antiviral Therapy for Chronic Hepatitis C. Mediat. Inflamm..

[13] Calmy A., Hirschel B., Cooper D.A., Carr A. (2007). Clinical Update: Adverse Effects of Antiretroviral Therapy. Lancet.

[14] Patra J.K., Das G., Fraceto L.F., Campos E.V.R., Rodriguez-Torres M.D.P., Acosta-Torres L.S., Diaz-Torres L.A., Grillo R., Swamy M.K., Sharma S. (2018). Nano Based Drug Delivery Systems: Recent Developments and Future Prospects. J. Nanobiotechnol.

[15] Vazquez-Muñoz R., Borrego B., Juárez-Moreno K., García-García M., Morales J.D.M., Bogdanchikova N., Huerta-Saquero A. (2017). Toxicity of Silver Nanoparticles in Biological Systems: Does the Complexity of Biological Systems Matter?. Toxicol. Lett..

[16] Telesnitsky A. (2010). Retroviruses: Molecular Biology, Genomics and Pathogenesis. Future Virol..

[17] Weiss R.A. (2013). On the Concept and Elucidation of Endogenous Retroviruses. Phil. Trans. R. Soc. B.

[18] Amara A., Littman D.R. (2003). After Hrs with HIV. J. Cell Biol..

[19] Perez O.D., Nolan G.P. (2001). Resistance Is Futile: Assimilation of Cellular Machinery by HIV-1. Immunity.

[20] Sharma S., Miyanohara A., Friedmann T. (2000). Separable Mechanisms of Attachment and Cell Uptake during Retrovirus Infection. J. Virol..

[21] Ugolini S., Mondor I., Sattentau Q.J. (1999). HIV-1 Attachment: Another Look. Trends Microbiol..

[22] Mondor I., Ugolini S., Sattentau Q.J. (1998). Human Immunodeficiency Virus Type 1 Attachment to HeLa CD4 Cells Is CD4 Independent and Gp120 Dependent and Requires Cell Surface Heparans. J. Virol..

[23] Hong P.W.-P., Flummerfelt K.B., De Parseval A., Gurney K., Elder J.H., Lee B. (2002). Human Immunodeficiency Virus Envelope (Gp120) Binding to DC-SIGN and Primary Dendritic Cells Is Carbohydrate Dependent but Does Not Involve 2G12 or Cyanovirin Binding Sites: Implications for Structural Analyses of Gp120-DC-SIGN Binding. J. Virol..

[24] Manel N., Kim F.J., Kinet S., Taylor N., Sitbon M., Battini J.-L. (2003). The Ubiquitous Glucose Transporter GLUT-1 Is a Receptor for HTLV. Cell.

[25] Maddon P.J., Dalgleish A.G., McDougal J.S., Clapham P.R., Weiss R.A., Axel R. (1986). The T4 Gene Encodes the AIDS Virus Receptor and Is Expressed in the Immune System and the Brain. Cell.

[26] Sherman M.P., Greene W.C. (2002). Slipping through the Door: HIV Entry into the Nucleus. Microbes Infect..

[27] Gallay P., Hope T., Chin D., Trono D. (1997). HIV-1 Infection of Nondividing Cells through the Recognition of Integrase by the Importin/Karyopherin Pathway. Proc. Natl. Acad. Sci. USA.

[28] Pryciak P.M., Sil A., Varmus H.E. (1992). Retroviral Integration into Minichromosomes in Vitro. EMBO J..

[29] De Clercq E. (2002). Strategies in the Design of Antiviral Drugs. Nat. Rev. Drug Discov..

[30] Lang L. (2009). FDA Grants Tentative Approval for 75th Generic Antiretroviral Drug. Gastroenterology.

[31] Walensky R.P., Paltiel A.D., Losina E., Mercincavage L.M., Schackman B.R., Sax P.E., Weinstein M.C., Freedberg K.A. (2006). The Survival Benefits of AIDS Treatment in the United States. J. Infect. Dis..

[32] Richman D.D., Margolis D.M., Delaney M., Greene W.C., Hazuda D., Pomerantz R.J. (2009). The Challenge of Finding a Cure for HIV Infection. Science.

[33] Richman D.D., Morton S.C., Wrin T., Hellmann N., Berry S., Shapiro M.F., Bozzette S.A. (2004). The Prevalence of Antiretroviral Drug Resistance in the United States. AIDS.

[34] das Neves J., Amiji M.M., Bahia M.F., Sarmento B. (2010). Nanotechnology-Based Systems for the Treatment and Prevention of HIV/AIDS. Adv. Drug Deliv. Rev..

[35] Nayak D., Boxi A., Ashe S., Thathapudi N.C., Nayak B. (2017). Stavudine Loaded Gelatin Liposomes for HIV Therapy: Preparation, Characterization and in Vitro Cytotoxic Evaluation. Mater. Sci. Eng. C.

[36] Javan F., Vatanara A., Azadmanesh K., Nabi-Meibodi M., Shakouri M. (2017). Encapsulation of Ritonavir in Solid Lipid Nanoparticles: In-Vitro Anti-HIV-1 Activity Using Lentiviral Particles. J. Pharm. Pharmacol..

[37] Leporati A., Novikov M.S., Valuev-Elliston V.T., Korolev S.P., Khandazhinskaya A.L., Kochetkov S.N., Gupta S., Goding J., Bolotin E., Gottikh M.B. (2016). Hydrophobic-Core PEGylated Graft Copolymer-Stabilized Nanoparticles Composed of Insoluble Non-Nucleoside Reverse Transcriptase Inhibitors Exhibit Strong Anti-HIV Activity. Nanomed. Nanotechnol. Biol. Med..

[38] Mandal S., Prathipati P.K., Belshan M., Destache C.J. (2019). A Potential Long-Acting Bictegravir Loaded Nano-Drug Delivery System for HIV-1 Infection: A Proof-of-Concept Study. Antivir. Res..

[39] Mandal S., Kang G., Prathipati P.K., Zhou Y., Fan W., Li Q., Destache C.J. (2019). Nanoencapsulation Introduces Long-Acting Phenomenon to Tenofovir Alafenamide and Emtricitabine Drug Combination: A Comparative Pre-Exposure Prophylaxis Efficacy Study against HIV-1 Vaginal Transmission. J. Control. Release.

[40] Mandal S., Kang G., Prathipati P.K., Fan W., Li Q., Destache C.J. (2018). Long-Acting Parenteral Combination Antiretroviral Loaded Nano-Drug Delivery System to Treat Chronic HIV-1 Infection: A Humanized Mouse Model Study. Antivir. Res..

[41] Fulcher J.A., Tamshen K., Wollenberg A.L., Kickhoefer V.A., Mrazek J., Elliott J., Ibarrondo F.J., Anton P.A., Rome L.H., Maynard H.D. (2019). Human Vault Nanoparticle Targeted Delivery of Antiretroviral Drugs to Inhibit Human Immunodeficiency Virus Type 1 Infection. Bioconjugate Chem..

[42] Zou X., Yuan M., Zhang T., Wei H., Xu S., Jiang N., Zheng N., Wu Z. (2019). Extracellular Vesicles Expressing a Single-Chain Variable Fragment of an HIV-1 Specific Antibody Selectively Target Env+ Tissues. Theranostics.

[43] Soleymani S., Yari F., Bolhassani A., Bakhshandeh H. (2019). Platelet Microparticles: An Effective Delivery System for Anti-Viral Drugs. J. Drug Deliv. Sci. Technol..

[44] Dou H., Grotepas C.B., McMillan J.M., Destache C.J., Chaubal M., Werling J., Kipp J., Rabinow B., Gendelman H.E. (2009). Macrophage Delivery of Nanoformulated Antiretroviral Drug to the Brain in a Murine Model of neuroAIDS. J. Immunol..

[45] Zhuang J., Gong H., Zhou J., Zhang Q., Gao W., Fang R.H., Zhang L. (2020). Targeted Gene Silencing in Vivo by Platelet Membrane–Coated Metal-Organic Framework Nanoparticles. Sci. Adv..

[46] Bolhassani A., Milani A. (2020). Small Interfering RNAs and Their Delivery Systems: A Novel Powerful Tool for the Potential Treatment of HIV Infections. Curr. Mol. Pharmacol..

[47] Rodriguez M., Lapierre J., Ojha C.R., Kaushik A., Batrakova E., Kashanchi F., Dever S.M., Nair M., El-Hage N. (2017). Intranasal Drug Delivery of Small Interfering RNA Targeting Beclin1 Encapsulated with Polyethylenimine (PEI) in Mouse Brain to Achieve HIV Attenuation. Sci. Rep..

[48] Rodriguez M., Kaushik A., Lapierre J., Dever S.M., El-Hage N., Nair M. (2017). Electro-Magnetic Nano-Particle Bound Beclin1 siRNA Crosses the Blood–Brain Barrier to Attenuate the Inflammatory Effects of HIV-1 Infection In Vitro. J. Neuroimmune Pharmacol..

[49] Gong Y., Chowdhury P., Nagesh P.K., Rahman M.A., Zhi K., Yallapu M.M., Kumar S. (2020). Novel Elvitegravir Nanoformulation for Drug Delivery across the Blood-Brain Barrier to Achieve HIV-1 Suppression in the CNS Macrophages. Sci. Rep..

[50] Ramanathan R., Jiang Y., Read B., Golan-Paz S., Woodrow K.A. (2016). Biophysical Characterization of Small Molecule Antiviral-Loaded Nanolipogels for HIV-1 Chemoprophylaxis and Topical Mucosal Application. Acta Biomater..

[51] Tomitaka A., Arami H., Huang Z., Raymond A., Rodriguez E., Cai Y., Febo M., Takemura Y., Nair M. (2018). Hybrid Magneto-Plasmonic Liposomes for Multimodal Image-Guided and Brain-Targeted HIV Treatment. Nanoscale.

[52] Dutta T., Agashe H.B., Garg M., Balasubramanium P., Kabra M., Jain N.K. (2007). Poly (Propyleneimine) Dendrimer Based Nanocontainers for Targeting of Efavirenz to Human Monocytes/Macrophages in Vitro: Research Paper. J. Drug Target..

[53] Dutta T., Jain N.K. (2007). Targeting Potential and Anti-HIV Activity of Lamivudine Loaded Mannosylated Poly (Propyleneimine) Dendrimer. Biochim. Et. Biophys. Acta (BBA) Gen. Subj..

[54] Dutta T., Garg M., Jain N.K. (2008). Targeting of Efavirenz Loaded Tuftsin Conjugated Poly (Propyleneimine) Dendrimers to HIV Infected Macrophages in Vitro. Eur. J. Pharm. Sci..

[55] Zhang G., Luk B.T., Wei X., Campbell G.R., Fang R.H., Zhang L., Spector S.A. (2019). Selective Cell Death of Latently HIV-Infected CD4+ T Cells Mediated by Autosis Inducing Nanopeptides. Cell Death Dis..

[56] Cao S., Slack S.D., Levy C.N., Hughes S.M., Jiang Y., Yogodzinski C., Roychoudhury P., Jerome K.R., Schiffer J.T., Hladik F. (2019). Hybrid Nanocarriers Incorporating Mechanistically Distinct Drugs for Lymphatic CD4^+^ T Cell Activation and HIV-1 Latency Reversal. Sci. Adv..

[57] Kumar P., Lakshmi Y.S., Golla K., Kondapi A.K. (2015). Improved Safety, Bioavailability and Pharmacokinetics of Zidovudine through Lactoferrin Nanoparticles during Oral Administration in Rats. PLoS ONE.

[58] Pan S., Weng H., Hu G., Wang S., Zhao T., Yao X., Liao L., Zhu X., Ge Y. (2021). Lactoferrin May Inhibit the Development of Cancer via Its Immunostimulatory and Immunomodulatory Activities. Int. J. Oncol..

[59] Tavakoli A., Rezaei F., Nasab G.S.F., Adjaminezhad-Fard F., Noroozbabaei Z., Mokhtari-Azad T. (2017). The Comparison of Sensitivity and Specificity of ELISA-Based Microneutralization Test with Hemagglutination Inhibition Test to Evaluate Neutralizing Antibody against Influenza Virus (H1N1). Iran. J. Public Health.

[60] Toledo-Rueda W., Rosas-Murrieta N.H., Muñoz-Medina J.E., González-Bonilla C., Reyes-Leyva J., Santos-López G. (2018). Antiviral Resistance Markers in Influenza Virus Sequences in Mexico, 2000–2017. Infect. Drug Resist..

[61] Levina A.S., Repkova M.N., Mazurkova N.A., Zarytova V.F. (2016). Nanoparticle-Mediated Nonviral DNA Delivery for Effective Inhibition of Influenza a Viruses in Cells. IEEE Trans. Nanotechnol..

[62] Li Y., Lin Z., Zhao M., Xu T., Wang C., Hua L., Wang H., Xia H., Zhu B. (2016). Silver Nanoparticle Based Codelivery of Oseltamivir to Inhibit the Activity of the H1N1 Influenza Virus through ROS-Mediated Signaling Pathways. ACS Appl. Mater. Interfaces.

[63] Chun H., Yeom M., Kim H.-O., Lim J.-W., Na W., Park G., Park C., Kang A., Yun D., Kim J. (2018). Efficient Antiviral Co-Delivery Using Polymersomes by Controlling the Surface Density of Cell-Targeting Groups for Influenza A Virus Treatment. Polym. Chem..

[64] Hu C.-M.J., Chen Y.-T., Fang Z.-S., Chang W.-S., Chen H.-W. (2018). Antiviral Efficacy of Nanoparticulate Vacuolar ATPase Inhibitors against Influenza Virus Infection. Int. J. Nanomed..

[65] Ghaffari H., Tavakoli A., Moradi A., Tabarraei A., Bokharaei-Salim F., Zahmatkeshan M., Farahmand M., Javanmard D., Kiani S.J., Esghaei M. (2019). Inhibition of H1N1 Influenza Virus Infection by Zinc Oxide Nanoparticles: Another Emerging Application of Nanomedicine. J. Biomed. Sci..

[66] Frede A., Neuhaus B., Knuschke T., Wadwa M., Kollenda S., Klopfleisch R., Hansen W., Buer J., Bruder D., Epple M. (2017). Local Delivery of siRNA-Loaded Calcium Phosphate Nanoparticles Abates Pulmonary Inflammation. Nanomed. Nanotechnol. Biol. Med..

[67] Jamali A., Mottaghitalab F., Abdoli A., Dinarvand M., Esmailie A., Kheiri M.T., Atyabi F. (2018). Inhibiting Influenza Virus Replication and Inducing Protection against Lethal Influenza Virus Challenge through Chitosan Nanoparticles Loaded by siRNA. Drug Deliv. Transl. Res..

[68] Timin A.S., Muslimov A.R., Petrova A.V., Lepik K.V., Okilova M.V., Vasin A.V., Afanasyev B.V., Sukhorukov G.B. (2017). Hybrid Inorganic-Organic Capsules for Efficient Intracellular Delivery of Novel siRNAs against Influenza A (H1N1) Virus Infection. Sci. Rep..

[69] Zhang C., Ren W., Liu Q., Tan Z., Li J., Tong C. (2019). Transportan-Derived Cell-Penetrating Peptide Delivers siRNA to Inhibit Replication of Influenza Virus in Vivo. Drug Des. Dev. Ther..

[70] Yutaka T., Masao M., Gallo R. (2019). 40 Years of the Human T-Cell Leukemia Virus: Past, Present, and Future. F1000Research.

[71] Robert-Guroff M., Safai B., Gelmann E., Mansell P.A., Groopman J., Sidhu G., Friedman-Kien A., Bayley A., Blayney D., Lange M. (1984). HTLV-I-Specific Antibody in AIDS Patients and Others at Risk. Lancet.

[72] Yamada Y., Tomonaga M., Fukuda H., Hanada S., Utsunomiya A., Tara M., Sano M., Ikeda S., Takatsuki K., Kozuru M. (2001). A New G-CSF-supported Combination Chemotherapy, LSG15, for Adult T-cell Leukaemia-lymphoma: Japan Clinical Oncology Group Study 9303. Br. J. Haematol..

[73] Tobinai K., Uike N., Saburi Y., Chou T., Etoh T., Masuda M., Kawano F., Matsuoka M., Taguchi H., Makino T. (2003). Phase II Study of Cladribine (2-Chlorodeoxyadenosine) in Relapsed or Refractory Adult T-Cell Leukemia-Lymphoma. Int. J. Hematol..

[74] O’Connor O.A., Pro B., Pinter-Brown L., Bartlett N., Popplewell L., Coiffier B., Lechowicz M.J., Savage K.J., Shustov A.R., Gisselbrecht C. (2011). Pralatrexate in Patients with Relapsed or Refractory Peripheral T-Cell Lymphoma: Results from the Pivotal PROPEL Study. J. Clin. Oncol..

[75] Amirnasr M., Sankian M., Rezaei A., Tafaghodi M. (2016). Immunization against HTLV-I with Chitosan and Tri-Methylchitosan Nanoparticles Loaded with Recombinant Env23 and Env13 Antigens of Envelope Protein Gp46. Microb. Pathog..

[76] Hekmat S., Aslani M.M., Shafiee Ardestani M., Aghasadeghi M.R., Siadat S.D., Sadat S.M., Mahdavi M., Shahbazi S., Asgarhalvaee F., Ghahari S.M.M. (2017). Preparation and Characterization of PLGA Nanoparticles Containing Recombinant Core-NS3 Fusion Protein of Hepatitis C Virus as a Nano-Vaccine Candidate. Vaccine Res..

[77] Ishihara T., Kaneko K., Ishihara T., Mizushima T. (2014). Development of Biodegradable Nanoparticles for Liver-Specific Ribavirin Delivery. J. Pharm. Sci..

[78] Lee M.-Y., Yang J.-A., Jung H.S., Beack S., Choi J.E., Hur W., Koo H., Kim K., Yoon S.K., Hahn S.K. (2012). Hyaluronic Acid–Gold Nanoparticle/Interferon α Complex for Targeted Treatment of Hepatitis C Virus Infection. ACS Nano.

[79] Kaneko K., Ishihara T. (2017). Development of Liver-Specific Ribavirin-Loaded Nanoparticles with Reduced Cytotoxicity. Cogent Med..

[80] Duan L., Yan Y., Liu J., Wang B., Li P., Hu Q., Chen W. (2016). Target Delivery of Small Interfering RNAs with Vitamin E-Coupled Nanoparticles for Treating Hepatitis C. Sci. Rep..

[81] Chen Y.-N., Hsu S.-L., Liao M.-Y., Liu Y.-T., Lai C.-H., Chen J.-F., Nguyen M.-H.T., Su Y.-H., Chen S.-T., Wu L.-C. (2017). Ameliorative Effect of Curcumin-Encapsulated Hyaluronic Acid–PLA Nanoparticles on Thioacetamide-Induced Murine Hepatic Fibrosis. Int. J. Environ. Res. Public. Health.

[82] Brochot E., Castelain S., Duverlie G., Capron D., Nguyen-Khac E., François C. (2010). Ribavirin Monitoring in Chronic Hepatitis C Therapy: Anaemia versus Efficacy. Antivir. Ther..

[83] Abo-Zeid Y.M., Urbanowicz R.A., Tarr A.W., Irving W.L., Thomson B.J., Garnett M.C. (2014). Nanoparticles as a Promising Delivery System to Improve Hepatitis C Treatment. https://www.semanticscholar.org/paper/Nanoparticles-as-a-Promising-Delivery-System-to-C-Abo-Zeid-Urbanowicz/653ba60c026716b8f040cefa70f86ceedfb037b4.

[84] Craparo E.F., Teresi G., Licciardi M., Bondí M.L., Cavallaro G. (2013). Novel Composed Galactosylated Nanodevices Containing a Ribavirin Prodrug as Hepatic Cell-Targeted Carriers for HCV Treatment. J. Biomed. Nanotechnol..

[85] Wang Z., Liu H., Yang S.H., Wang T., Liu C., Cao Y.C. (2012). Nanoparticle-Based Artificial RNA Silencing Machinery for Antiviral Therapy. Proc. Natl. Acad. Sci. USA.

[86] Khaliq S., Raza S.M. (2018). Current Status of Direct Acting Antiviral Agents against Hepatitis C Virus Infection in Pakistan. Medicina.

[87] Gonzales Zamora J.A. (2018). Adverse Effects of Direct Acting Antivirals in HIV/HCV Coinfected Patients: A 4-Year Experience in Miami, Florida. Diseases.

[88] Shand H., Dutta S., Rajakumar S., James Paulraj S., Mandal A.K., KT R.D., Ghorai S. (2022). New Age Detection of Viruses: The Nano-Biosensors. Front. Nanotechnol..

[89] Abdullahi I.N., Emeribe A.U., Mustapha J.O., Fasogbon S.A., Ofor I.B., Opeyemi I.S., Obi-George C., Sunday A.O., Nwofe J. (2020). Exploring the Genetics, Ecology of SARS-CoV-2 and Climatic Factors as Possible Control Strategies against COVID-19. Infez. Med..

[90] Abd Ellah N.H., Gad S.F., Muhammad K., Batiha G.E., Hetta H.F. (2020). Nanomedicine as a Promising Approach for Diagnosis, Treatment and Prophylaxis against COVID-19. Nanomedicine.

[91] Mainardes R.M., Diedrich C. (2020). The Potential Role of Nanomedicine on COVID-19 Therapeutics. Ther. Deliv..

[92] Huang J., Li Y., Orza A., Lu Q., Guo P., Wang L., Yang L., Mao H. (2016). Magnetic Nanoparticle Facilitated Drug Delivery for Cancer Therapy with Targeted and Image-Guided Approaches. Adv. Funct. Mater..

[93] Kaushik A., Jayant R.D., Sagar V., Nair M. (2014). The Potential of Magneto-Electric Nanocarriers for Drug Delivery. Expert. Opin. Drug Deliv..

[94] De Wilde A.H., Snijder E.J., Kikkert M., Van Hemert M.J., Tripp R.A., Tompkins S.M. (2017). Host Factors in Coronavirus Replication. Roles of Host Gene and Non-Coding RNA Expression in Virus Infection.

[95] Liu J., Wan M., Lyon C.J., Hu T.Y. (2020). Nanomedicine Therapies Modulating Macrophage Dysfunction: A Potential Strategy to Attenuate Cytokine Storms in Severe Infections. Theranostics.

[96] He H., Ghosh S., Yang H. (2017). Nanomedicines for Dysfunctional Macrophage-Associated Diseases. J. Control. Release.

[97] Zare M., Thomas V., Ramakrishna S. (2021). Nanoscience and Quantum Science-Led Biocidal and Antiviral Strategies. J. Mater. Chem. B.

[98] Shand H., Dutta S., Patra S., Jain H., Mondal R., Mandal A.K., Ghorai S. (2024). Nanoparticle-Based Intervention to Cardiovascular Diseases (CVDS). Appl. Nanosci..

[99] Jangra S., Landers J.J., Rathnasinghe R., O’Konek J.J., Janczak K.W., Cascalho M., Kennedy A.A., Tai A.W., Baker J.R., Schotsaert M. (2021). A Combination Adjuvant for the Induction of Potent Antiviral Immune Responses for a Recombinant SARS-CoV-2 Protein Vaccine. Front. Immunol..

[100] Mamo T., Poland G.A. (2012). Nanovaccinology: The next Generation of Vaccines Meets 21st Century Materials Science and Engineering. Vaccine.

[101] Sakaue G., Hiroi T., Nakagawa Y., Someya K., Iwatani K., Sawa Y., Takahashi H., Honda M., Kunisawa J., Kiyono H. (2003). HIV Mucosal Vaccine: Nasal Immunization with Gp160-Encapsulated Hemagglutinating Virus of Japan-Liposome Induces Antigen-Specific CTLs and Neutralizing Antibody Responses. J. Immunol..

[102] Letvin N.L. (2006). Progress and Obstacles in the Development of an AIDS Vaccine. Nat. Rev. Immunol..

[103] Rostami H., Ebtekar M., Ardestani M.S., Yazdi M.H., Mahdavi M. (2017). Co-Utilization of a TLR5 Agonist and Nano-Formulation of HIV-1 Vaccine Candidate Leads to Increased Vaccine Immunogenicity and Decreased Immunogenic Dose: A Preliminary Study. Immunol. Lett..

[104] Liu Y., Chen C. (2016). Role of Nanotechnology in HIV/AIDS Vaccine Development. Adv. Drug Deliv. Rev..

[105] Tőke E.R., Lőrincz O., Csiszovszki Z., Somogyi E., Felföldi G., Molnár L., Szipőcs R., Kolonics A., Malissen B., Lori F. (2014). Exploitation of Langerhans Cells for in Vivo DNA Vaccine Delivery into the Lymph Nodes. Gene Ther..

[106] Rioux G., Mathieu C., Russell A., Bolduc M., Laliberté-Gagné M.-E., Savard P., Leclerc D. (2014). PapMV Nanoparticles Improve Mucosal Immune Responses to the Trivalent Inactivated Flu Vaccine. J. Nanobiotechnol..

[107] Dehghan S., Tafaghodi M., Bolourieh T., Mazaheri V., Torabi A., Abnous K., Kheiri M.T. (2014). Rabbit Nasal Immunization against Influenza by Dry-Powder Form of Chitosan Nanospheres Encapsulated with Influenza Whole Virus and Adjuvants. Int. J. Pharm..

[108] Narasimhan B., Ross K., Loyd H., Wu W., Huntimer L., Ahmed S., Sambol A., Broderick S., Flickinger Z., Rajan K. (2014). Hemagglutinin-Based Polyanhydride Nanovaccines against H5N1 Influenza Elicit Protective Virus Neutralizing Titers and Cell-Mediated Immunity. Int. J. Nanomed..

[109] Walsh E.E., Frenck R.W., Falsey A.R., Kitchin N., Absalon J., Gurtman A., Lockhart S., Neuzil K., Mulligan M.J., Bailey R. (2020). Safety and Immunogenicity of Two RNA-Based COVID-19 Vaccine Candidates. N. Engl. J. Med..

[110] Granados-Riveron J.T., Aquino-Jarquin G. (2021). Engineering of the Current Nucleoside-Modified mRNA-LNP Vaccines against SARS-CoV-2. Biomed. Pharmacother..

[111] Kwiatkowska A., Granicka L.H. (2023). Anti-Viral Surfaces in the Fight against the Spread of Coronaviruses. Membranes.

[112] Tatlow D., Tatlow C., Tatlow S., Tatlow S. (2020). A novel concept for treatment and vaccination against COVID-19 with an inhaled chitosan-coated DNA vaccine encoding a secreted spike protein portion. Clin. Exp. Pharmacol. Physiol..

[113] Radema M.H., Van Eijk J.L., Vermin W., De Kok A.J., Romers C. (1979). Alkaloids of South African Samples of Calpurnia Aurea Subsp. Sylvatica. Phytochem..

[114] Kumar S.D., Singaravelu G., Ajithkumar S., Murugan K., Nicoletti M., Benelli G. (2017). Mangrove-Mediated Green Synthesis of Silver Nanoparticles with High HIV-1 Reverse Transcriptase Inhibitory Potential. J. Clust. Sci..

[115] Premanathan M., Kathiresan K., Nakashima H. (1999). Mangrove Halophytes: A Source of Antiviral Substances. South. Pac. Study.

[116] Rai M., Yadav A., Gade A. (2009). Silver Nanoparticles as a New Generation of Antimicrobials. Biotechnol. Adv..

[117] Yin Y., Wunderink R.G. (2018). MERS, SARS and Other Coronaviruses as Causes of Pneumonia. Respirology.

[118] Dube A., Nicolazzo J.A., Larson I. (2010). Chitosan Nanoparticles Enhance the Intestinal Absorption of the Green Tea Catechins (+)-Catechin and (−)-Epigallocatechin Gallate. Eur. J. Pharm. Sci..

[119] Sindhu R.K., Gupta R., Wadhera G., Kumar P. (2022). Modern Herbal Nanogels: Formulation, Delivery Methods, and Applications. Gels.

[120] Sims K.R., He B., Koo H., Benoit D.S.W. (2020). Electrostatic Interactions Enable Nanoparticle Delivery of the Flavonoid Myricetin. ACS Omega.

[121] Kumari A., Kumar V., Yadav S.K. (2012). Plant Extract Synthesized PLA Nanoparticles for Controlled and Sustained Release of Quercetin: A Green Approach. PLoS ONE.

[122] Fiorani M., Accorsi A., Cantoni O. (2003). Human Red Blood Cells as A Natural Flavonoid Reservoir. Free Radic. Res..

[123] Zhang H., Yang X., Zhao L., Jiao Y., Liu J., Zhai G. (2015). In Vitro and in Vivo Study of Baicalin-Loaded Mixed Micelles for Oral Delivery. Drug Deliv..

[124] Dokania S., Joshi A.K. (2015). Self-Microemulsifying Drug Delivery System (SMEDDS)—Challenges and Road Ahead. Drug Deliv..

[125] Feng R., Zhang Z., Li Z., Huang G. (2014). Preparation and in Vitro Evaluation of Etoposide-Loaded PLGA Microspheres for Pulmonary Drug Delivery. Drug Deliv..

[126] Yue P.-F., Yuan H.-L., Xie H., Xiao X.-H., Yang M., Liao M.-X., Zhu W.-F., Cai P.-L. (2008). Preparation, Characterization, and Bioavailability of Ursodeoxycholic Acid–Phospholipid Complex In Vivo. Drug Dev. Ind. Pharm..

[127] Al-Sanea M.M., Abelyan N., Abdelgawad M.A., Musa A., Ghoneim M.M., Al-Warhi T., Aljaeed N., Alotaibi O.J., Alnusaire T.S., Abdelwahab S.F. (2021). Strawberry and Ginger Silver Nanoparticles as Potential Inhibitors for SARS-CoV-2 Assisted by in Silico Modeling and Metabolic Profiling. Antibiotics.

[128] Kurniawan D.W., Ikhsanudin A. (2020). Potential of Jamu in Nanotechnology Perspective as an Alternative Treatment for COVID-19. Pharm. Sci. Res..

[129] Yang R., Huang X., Dou J., Zhai G., Su L. (2013). Self-Microemulsifying Drug Delivery System for Improved Oral Bioavailability of Oleanolic Acid: Design and Evaluation. Int. J. Nanomed..

[130] Etman S.M., Elnaggar Y.S., Abdallah O.Y. (2020). Fucoidan, a Natural Biopolymer in Cancer Combating: From Edible Algae to Nanocarrier Tailoring. Int. J. Biol. Macromol..

[131] Gholami M., Adibipour F., Valipour S.M., Ulloa L., Motaghinejad M. (2022). Potential Regulation of NF-κB by Curcumin in Coronavirus-Induced Cytokine Storm and Lung Injury. Int. J. Prev. Med..

[132] Ahmadi R., Salari S., Sharifi M.D., Reihani H., Rostamiani M.B., Behmadi M., Taherzadeh Z., Eslami S., Rezayat S.M., Jaafari M.R. (2021). Oral Nano-curcumin Formulation Efficacy in the Management of Mild to Moderate Outpatient COVID-19: A Randomized Triple-blind Placebo-controlled Clinical Trial. Food Sci. Nutr..

[133] Yang J., Jia C., Yang J. (2021). Designing Nanoparticle-Based Drug Delivery Systems for Precision Medicine. Int. J. Med. Sci..

[134] Lv X., Wang P., Bai R., Cong Y., Suo S., Ren X., Chen C. (2014). Inhibitory Effect of Silver Nanomaterials on Transmissible Virus-Induced Host Cell Infections. Biomaterials.

[135] Chen Y.-N., Hsueh Y.-H., Hsieh C.-T., Tzou D.-Y., Chang P.-L. (2016). Antiviral Activity of Graphene–Silver Nanocomposites against Non-Enveloped and Enveloped Viruses. Int. J. Environ. Res. Public. Health.

[136] Kandeel M., Al-Taher A., Park B.K., Kwon H., Al-Nazawi M. (2020). A Pilot Study of the Antiviral Activity of Anionic and Cationic Polyamidoamine Dendrimers against the Middle East Respiratory Syndrome Coronavirus. J. Med. Virol..

[137] Lin Z., Wang Z., Zhang X., Diao D. (2021). Superhydrophobic, Photo-Sterilize, and Reusable Mask Based on Graphene Nanosheet-Embedded Carbon (GNEC) Film. Nano Res..

[138] Sharifi-Rad J., Quispe C., Rahavian A., Pereira Carneiro J.N., Rocha J.E., Alves Borges Leal A.L., Bezerra Morais Braga M.F., Melo Coutinho H.D., Ansari Djafari A., Alarcón-Zapata P. (2021). Bioactive Compounds as Potential Agents for Sexually Transmitted Diseases Management: A Review to Explore Molecular Mechanisms of Action. Front. Pharmacol..

